# Emergence and Molecular Epidemiology of Campylobacter jejuni ST-2993 Associated with a Large Outbreak of Guillain-Barré Syndrome in Peru

**DOI:** 10.1128/spectrum.01187-22

**Published:** 2022-08-16

**Authors:** Willi Quino, Junior Caro-Castro, Orson Mestanza, Verónica Hurtado, María Luz Zamudio, Gloria Cruz-Gonzales, Ronnie G. Gavilan

**Affiliations:** a Laboratorio de Referencia Nacional de Enteropatógenos, Instituto Nacional de Salud, Lima, Perú; b Escuela Universitaria de Posgrado, Universidad Nacional Federico Villarreal, Lima, Perú; c Escuela Profesional de Medicina Humana, Universidad Privada San Juan Bautista, Lima, Perú; University of Tennessee

**Keywords:** *Campylobacter jejuni*, disease outbreaks, Guillain-Barré syndrome, molecular typing, phylogenomics

## Abstract

Campylobacter jejuni infection is considered the most frequent factor associated with Guillain-Barré syndrome (GBS). In 2019, a large outbreak of GBS was detected in Peru, being associated with C. jejuni detected in stool samples from these patients. The aim of this study was to determine the molecular epidemiology of C. jejuni strains (ST-2993) associated with a large GBS outbreak in Peru. In this study, 26 C. jejuni strains belonging to the ST-2293, obtained from 2019 to 2020, were sequenced using Illumina technology. Five low-quality sequences were removed using bioinformatics, and 21 genomes (17 clinical strains and 4 chicken strains) were considered in the phylogenetic analysis and comparative genomics. Phylogenetic reconstruction, including genomes from international databases, showed a connection between Peruvian and Chinese GBS strains, both of them having lipooligosaccharides (LOS) locus genes related to molecular mimicry with gangliosides in peripheral nerves. Also, ST-2993 was detected in Amazon strains recovered many years before the 2019 outbreak, but with no epidemiological connection with GBS. Besides, a close relationship between human and chicken C. jejuni strains indicated chicken as one of the probable reservoirs. Finally, comparative genomics revealed differences between Chinese and Peruvian strains, including the presence of a prophage inserted into the genome. In conclusion, C. jejuni ST-2993 strains recovered from the GBS outbreak are closely related to Peruvian Amazon strains. Moreover, ST-2993 has been circulated in Peru since 2003 in the Peruvian Amazonia, showing the necessity to reinforce the epidemiological surveillance of C. jejuni to improve the prevention and control of future GBS outbreaks.

**IMPORTANCE** This article describes the molecular epidemiology of C. jejuni strains (ST-2993) associated with a large Guillain-Barré Syndrome (GBS) outbreak in Peru, sequencing several strains recovered from GBS patients and chickens from 2019 to 2020. Phylogenetic analysis showed a connection between Peruvian and Chinese GBS strains, both of them having lipooligosaccharides (LOS) locus genes related to molecular mimicry with gangliosides in peripheral nerves. Also, ST-2993 strains were detected in isolates recovered many years before the 2019 outbreak, but with no epidemiological connection with GBS. Besides, a close relationship between human and chicken strains indicated those animals as a probable reservoir. This information will help to understand the real situation of GBS in Peru and its causal agent, C. jejuni ST-2993, showing the necessity to increase epidemiological tracking of these kinds of pathogens to detect them and avoid GBS outbreaks in the future.

## INTRODUCTION

Guillain-Barré syndrome (GBS) is an autoimmune disorder of the peripheral nervous system (PNS), characterized by an acute or subacute symmetrical ascending motor weakness, dysreflexia, and mild to moderate sensory abnormalities, that has now become the most common cause of acute flaccid paralysis, with an approximate annual incidence of 0.6–4 cases per 100,000 people worldwide (1). Approximately 60% of patients with GBS have a previous episode of gastrointestinal or respiratory infection, caused from a bacterium or a virus, weeks before the onset of neurological symptoms (2). Among all the microorganisms that can trigger GBS, Campylobacter jejuni is the most frequent pathogen associated with GBS (26-41%), which usually causes gastroenteritis several weeks before the beginning of GBS symptoms (3).

After a C. jejuni infection in susceptible individuals, antibodies that react cross-linked with specific gangliosides are synthesized (4). Molecular mimicry between lipooligosaccharides (LOS) of the cell wall from certain subtypes of C. jejuni and gangliosides in peripheral nerves plays a crucial role in the pathogenesis of GBS. The acute phase sera of most patients with GBS associated with C. jejuni contain elevated titers of those antibodies against various gangliosides that cross-react with LOS of C. jejuni (5). The specificities of these antiganglioside antibodies are related to different clinical presentations of GBS: anti-GM1 antibodies have been associated with a pure and severe motor form of GBS (acute inflammatory demyelinating polyneuropathy, AIDP) (6), and anti-GD1a is associated with the axonal form (acute motor axonal neuropathy, AMAN) (7), while Miller Fisher syndrome (MFS), a variant of GBS with oculomotor weakness and ataxia, is strongly associated with anti-GQ1b antibodies (8). However, not all C. jejuni strains are involved in GBS cases worldwide, but are restricted to certain serotypes and genotypes of this bacterium. For example, using the Penner serotyping scheme, several GBS outbreaks were linked to C. jejuni HS:41 (9, 10). Besides, using genomics techniques like multilocus sequence type (MLST), it has been possible to determine some of them. These include ST-362, which has caused the most GBS cases in South Africa since 1994 (9), and ST-2993, which triggered an outbreak in Jilin province, China during 2007 (10), both genotypes belonging to the CC-362.

Recently, scientists learned about the genetic basis of C. jejuni related to GBS, using several molecular techniques, but there are still no effective tools available to trace the exact source of an outbreak caused by this microorganism. The currently used techniques to obtain DNA fingerprints of this microbial agent often cannot discriminate among all the bacterial strains of the same outbreak, making it difficult to follow the spread of this disease (11). In this context, the application of whole-genome sequencing (WGS) is proposed as a molecular tool, which allows determining all the available genetic information of each clinical isolate and establishing an association between the genomes that cause an outbreak and their possible source through small similarities and variants of their genomes.

In Peru, C. jejuni is one of the main etiological agents that cause watery diarrhea, mainly in children (12). This, added to microbial resistance previously reported (13), had already made it a highly important microorganism in public health. In early May 2019, several cases of suspected GBS were reported by the Peruvian Ministry of Health surveillance system, having a value that exceed the expected incidence, 29 cases per 100,000 people. Several samples like serum, urine, nasal swabs, and stool were tested using molecular panels for multipathogen detection, finding C. jejuni in stool samples (14). Genomic sequencing detected the same genotype (ST-2993) in several cases from different Peruvian regions (15), confirming the causal agent of GBS in this outbreak. In fact, this GBS Peruvian outbreak is considered one of the largest GBS outbreaks around the world, showing 305 cases in the 23th epidemiological week, the maximum peak of this outbreak (14). With the aim obtaining information about the genetic relationship between Peruvian C. jejuni strains involved in this large GBS outbreak, this study performed phylogenetic and comparative analysis between strains recovered in this study and other strains from related studies using several bioinformatic tools.

## RESULTS

### GBS outbreak in Peru.

**FIG 1:**
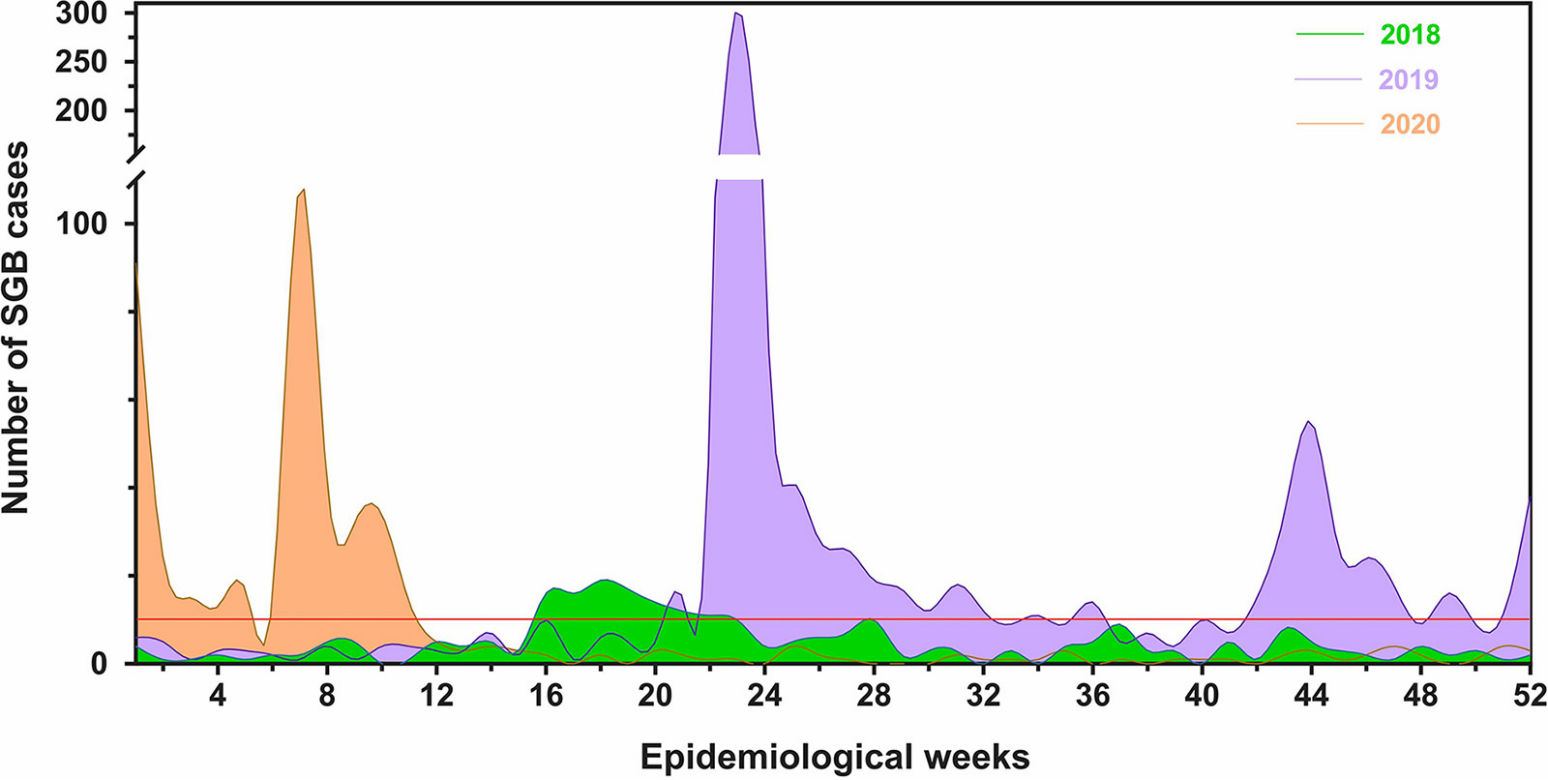
Guillain-Barré Syndrome cases from 2018 to 2020 in Peru. Each color indicates a different year. *x* axis indicates the epidemiological weeks, while *y* axis indicates number of GBS cases.

The total number of plotted GBS cases showed that the number of reported cases in 2019 and 2020 was higher than the cases of 2018. The maximum peak of cases in 2018 was detected in the 18th epidemiological week (May, *n* = 19), while the maximum peak of cases in 2019 and 2020 were detected in the 23th (June, *n* = 305) and 7th (February, *n* = 109) epidemiological weeks, respectively. Moreover, several epidemiological weeks of 2019 and 2020 reported more cases than the maximum peak of 2018 (Fig. 1).

### Microbiological characterization of C. jejuni strains.

**FIG 2:**
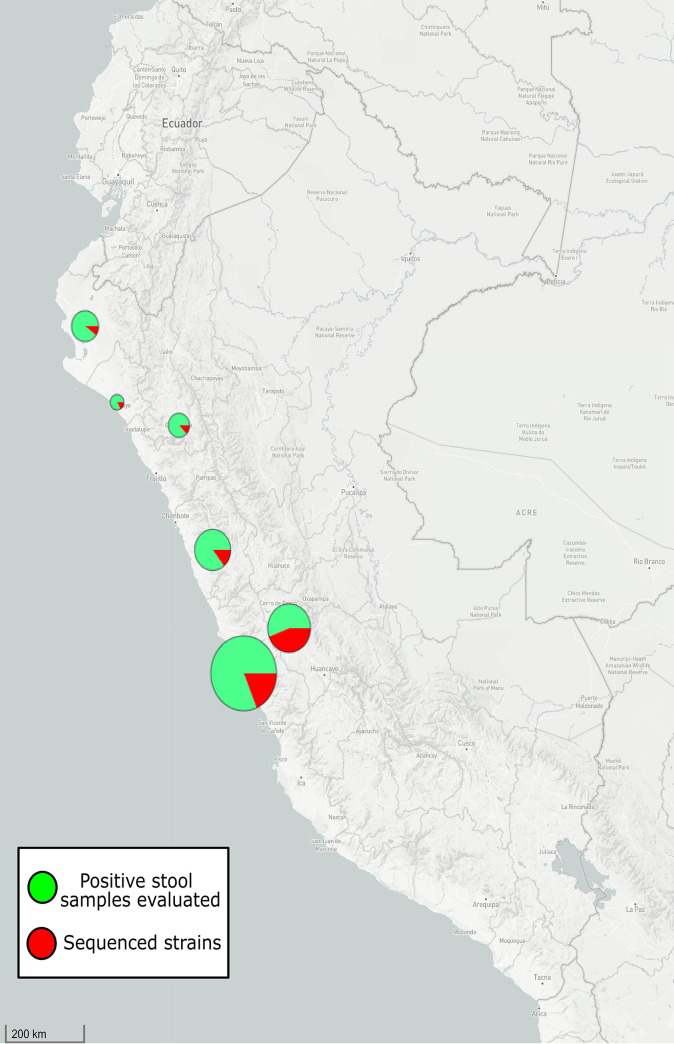
Map of Peru and geographical origin of positive stool samples for C. jejuni by conventional and automated PCR generated using the Microreact tool. The size of circular graphs indicates the proportion of isolates of each location. Color green indicates the total of samples, while color red indicates the samples in which C. jejuni strains could be isolated and sequenced.

From the 99 positive samples for C. jejuni, a total of 26 pure cultures were recovered (20 clinical and 6 chicken strains); all of them biotyped as C. jejuni biotype I using conventional microbiological procedures (positive hippurate hydrolysis, negative hydrogen sulfide production, and negative DNA hydrolysis) and confirmed by PCR. At least one clinical strain from the six Peruvian regions (Lima, Junin, La Libertad, Lambayeque, Cajamarca, and Piura) were recovered as pure culture (Table 1). On the other hand, chicken strains recovered from cloacal swabs came from broiler flocks located in Junin (Fig. 2).

**TABLE 1:** Total of positive stool samples evaluated in this study

Region	2019		2020		Total positive samples	Genome sequences
	Human	Chicken	Human	Chicken		
Cajamarca	7	0	1	0	8	1
Junin	8	0	3	7	18	8
La Libertad	4	0	10	0	14	2
Lambayeque	5	0	1	0	6	1
Lima	22	0	21	0	43	8
Piura	7	0	3	0	10	1
Total	53	0	39	7	99	21

### Genomic analysis and MLST of C. jejuni.

The evaluation of reads detected five isolates with low-quality information (3 clinical and 2 chicken strains) and were excluded from the analysis, getting a total of 21 strains (17 clinical and 4 chicken strains) to be evaluated (Table 2).

**TABLE 2:** Information of Peruvian C. jejuni ST-2993 isolates sequenced in this study

Strain	yr	Source	Illness	Country	City	SRA accession	NCBI assembly
6.897-2019	2019	Human	GBS	Peru	Piura	SRR20336842	GCA_021889995.1
1.1279-2019	2019	Human	GBS	Peru	Lima	SRR20336847	GCA_021890275.1
1.1280-2019	2019	Human	GBS	Peru	Lima	SRR20336846	GCA_021890255.1
1.1281-2019	2019	Human	GBS	Peru	Lima	SRR20336835	GCA_021890235.1
1.1282-2019	2019	Human	GBS	Peru	Lima	SRR20336833	GCA_021890215.1
6.1083-2019	2019	Human	GBS	Peru	Lambayeque	SRR20336831	GCA_021890175.1
6.1195-2019	2019	Human	GBS	Peru	Junín	SRR20336832	GCA_021890165.1
6.1196-2019	2019	Human	GBS	Peru	Junín	SRR20336830	GCA_021890155.1
6.1197-2019	2019	Human	GBS	Peru	Junín	SRR20336829	GCA_021890135.1
6.1198-2019	2019	Human	GBS	Peru	Junín	SRR20336828	GCA_021890115.1
6.2107-2019	2019	Human	GBS	Peru	La Libertad	SRR20336827	GCA_021890095.1
6.2108-2019	2019	Human	GBS	Peru	La Libertad	SRR20336845	GCA_021890075.1
6.2116-2019	2019	Human	GBS	Peru	Lima	SRR20336844	GCA_021890055.1
6.2139-2019	2019	Human	GBS	Peru	Cajamarca	SRR20336843	GCA_021890015.1
6.059-2020	2020	Human	GBS	Peru	Lima	SRR20336840	GCA_021889955.1
6.060-2020	2020	Human	GBS	Peru	Lima	SRR20336841	GCA_021889975.1
6.066-2020	2020	Human	GBS	Peru	Lima	SRR20336839	GCA_021889935.1
4-168-2020	2020	Chicken	Avian infection	Peru	Junín	SRR20336834	GCA_021889815.1
4.169-2020	2020	Chicken	Avian infection	Peru	Junín	SRR20336837	GCA_021889895.1
4.170-2020	2020	Chicken	Avian infection	Peru	Junín	SRR20336838	GCA_021889905.1
4.171-2020	2020	Chicken	Avian infection	Peru	Junín	SRR20336836	GCA_021889875.1

Genomic information obtained had on average 5.2 million reads. The number of contigs obtained after assembling was less than 30 for each genome, the G+C percentage was 30.14%, and the average size of obtained genomes was 1.6 Mpb. MLST determined that all 21 sequenced strains of C. jejuni corresponded to ST-2993: *aspA* (1), *glnA* (2), *gltA* (42), *glyA* (4), *pgm* (11), *tkt* (9), and *uncA* (8).

All the other 12 genomes recovered from previous studies (16–18) were reconfirmed as ST-2993 by MLST, making a total of 31 C. jejuni genomes for ST-2993 phylogenetic analysis.

The maximum-likelihood phylogeny of C. jejuni strains that represent all genetic diversity of this pathogen detected in Peru showed that all ST-2293 grouped together forming a strong cluster, with 100% of bootstrap inside cluster II, and having more than 0.0019 substitutions per site with the closest genotype, ST-3572 (Fig. S1).

### Phylogenomic analysis of C. jejuni ST-2293.

**FIG 3:**
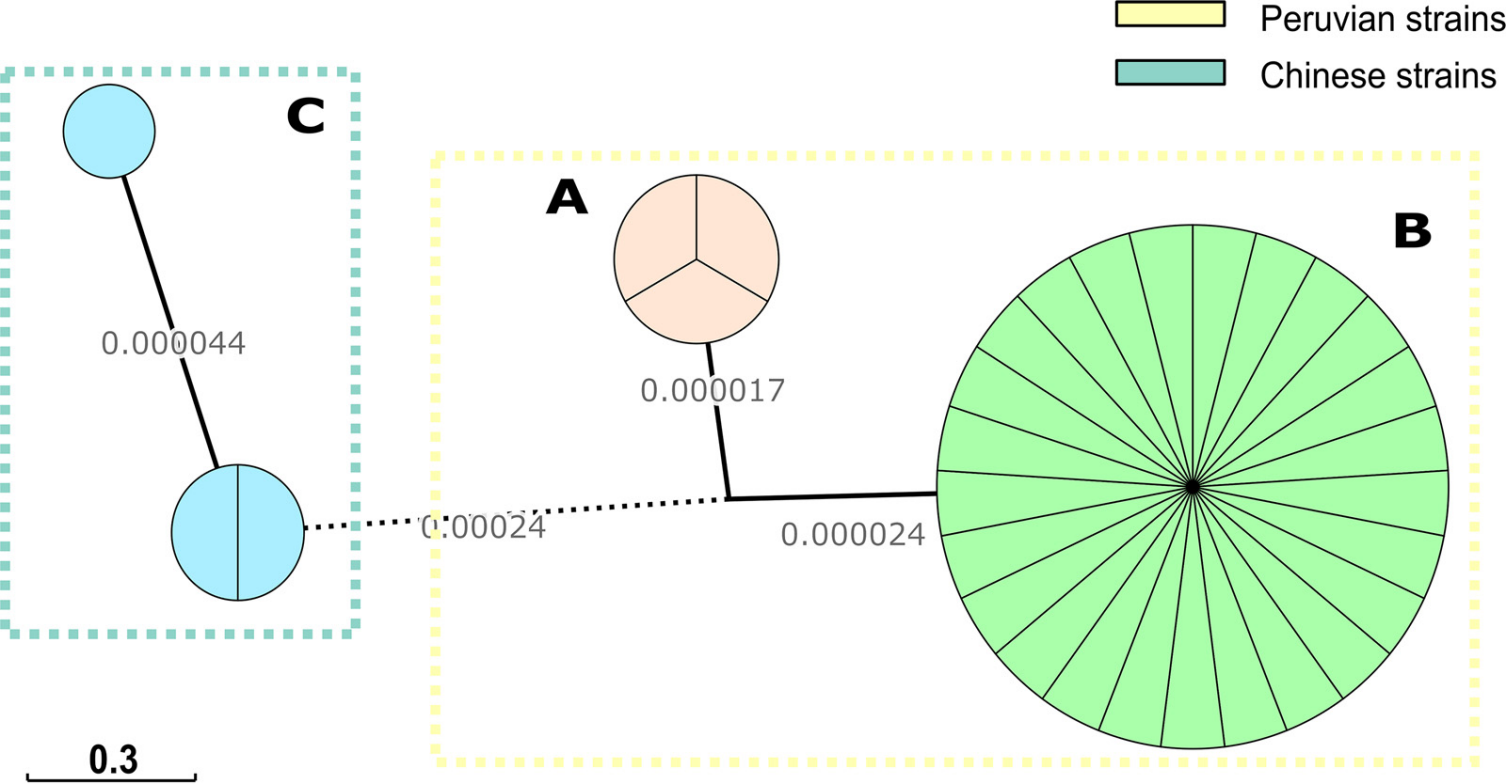
Whole-genome MST of C. jejuni ST-2993 used in this study. The numbers over branches infer relatedness by stating the substitution per sequence site. Branches spanning <0.00001 substitutions per site were collapsed for clarity. A represents Peruvian Amazon strains while B represents Peruvian GBS strains. C represents Chinese GBS strains.

The alignment using harvest tools presented 2,336 single nucleotide polymorphisms (SNPs) within locally colinear blocks of more than 1.5 Mb. The relationship of all isolates using a minimum spanning tree (MST) determined that there is a bigger distance between Chinese GBS strains and Peruvian GBS strains than between Peruvian Amazon strains and Peruvian GBS strains. Also, there is a close connection between Peruvian clinical GBS strains and chicken strains, forming a unique cluster (Fig. 3).

**FIG 4:**
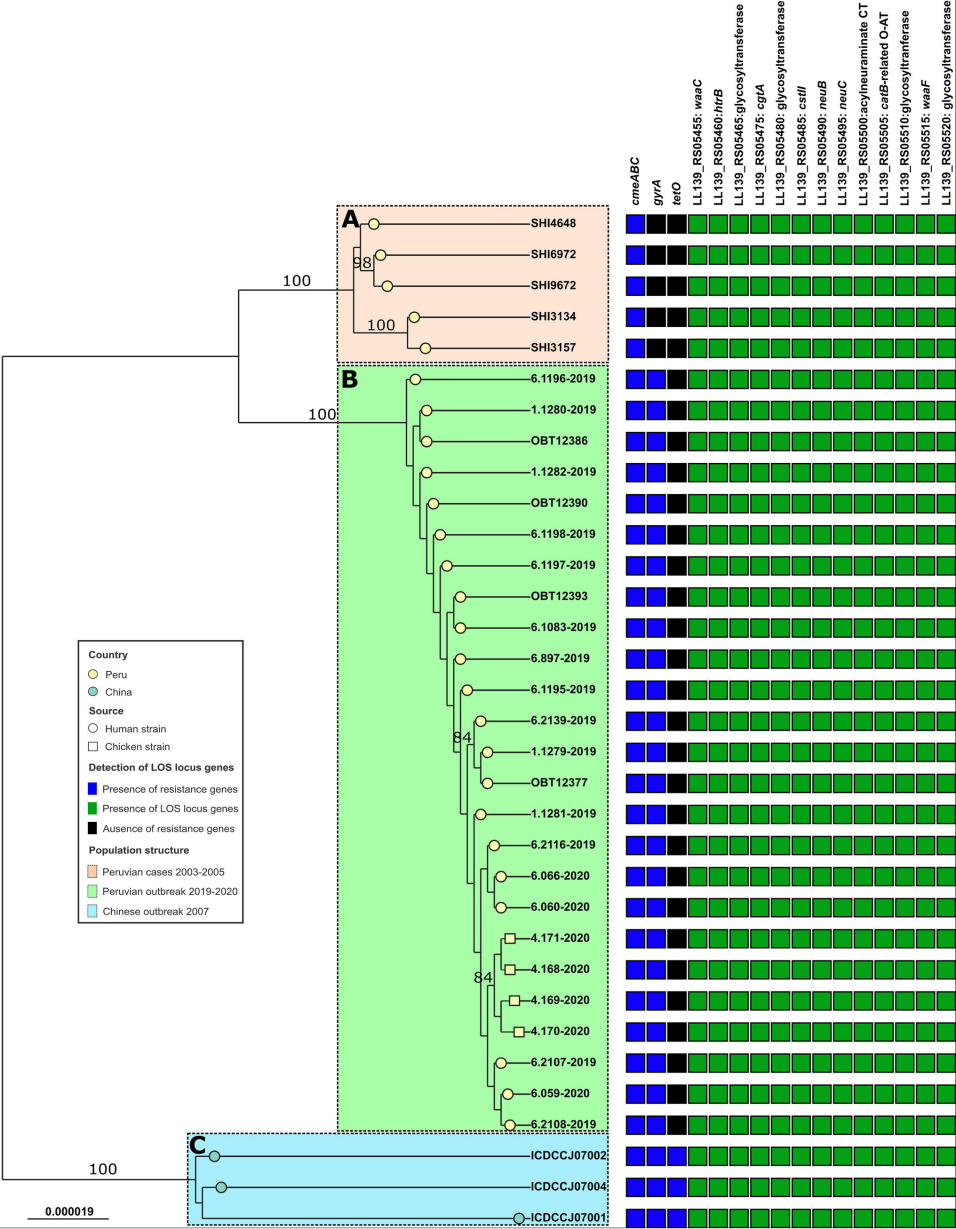
Core genome maximum-likelihood phylogeny of Peruvian and Chinese C. jejuni ST-2993 from human and chicken samples. Branch lengths indicate substitution per sequence site. Bootstrap values calculated over 1,000 repetitions are indicated at internal nodes. Only bootstrap values above 80% are displayed. Resistance genes such as *cmeABC*, *gyrA* mutation, and *tetO* were detected using the CARD database. LOS locus genes such as *htrB*, *cgtA*, *cstII*, *neuB*, and *neuC* genes were mapped using BLAST. The capital letters and color blocks indicate the detected clades: (A) Peruvian Amazon children’s strains, (B) Peruvian GBS (chicken and human) strains, and (C) Chinese GBS strains. Bar, 0.000019 substitution per sequence site.

Phylogenetic analysis revealed the ST-2993 descended from the same branch. The three Chinese strains are located in the most external branches (sky blue cluster); Peruvian Amazon strains are the most external ones related to Peruvian strains (pink cluster), and there is a close relationship between Peruvian GBS and chicken strains, but all isolates from this final group are grouped into the same cluster (light green cluster). Note that nodes from the principal clusters had a bootstrap of 100%. LOS locus genes were detected in all genomes evaluated. However, Peruvian Amazon strains lacked the *gyrA* mutation and only Chinese strains had the *tetO* gene encoded in a plasmid (Fig. 4).

### Pangenome of C. jejuni ST-2993.

**FIG 5:**
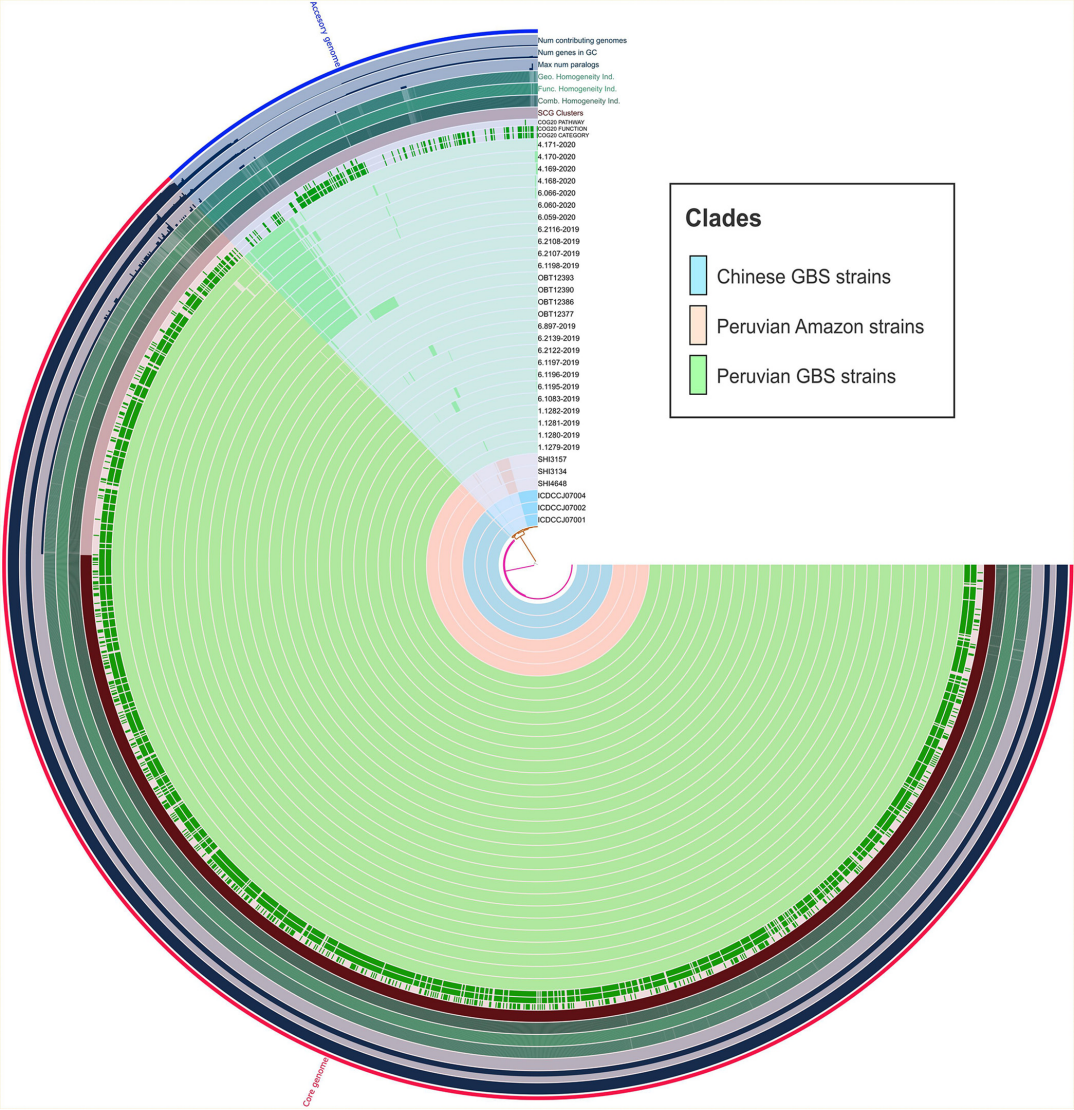
Comparative genomic analysis of C. jejuni ST-2993 genomes included in this study. The inner layers represent individual genomes organized regarding their phylogenetic relationships according to the maximum likelihood tree using the same colors. Sky blue: Chinese GBS strains. Pink: Peruvian Amazon strains not related to GBS. Light green: Peruvian GBS strains. In the layers, dark colors indicate the presence of a gene group and light colors its absence. The core and the accessory genomes are indicated in red and blue, respectively, in the outmost layer. The blue layers represent the number of genomes among the population contributing to each gene group, GC content of genes, and the number of paralogs, respectively; the dark green layers describe the genetic, functional, and combined homogeneity indexes, respectively. There is high homology inside Chinese GBS strains and Peruvian Amazon strains, being almost identical. Also, there are two visible groups inside Peruvian GBS strains, according to their accessory genes.

Pangenome analysis revealed high homology presented between ST-2993 genomes, being almost identical (Fig. 5), having a core genome of 94.8%. It was possible to note accessory genes inside Chinese genomes (sky blue) and Peruvian Amazon children C. jejuni (pink) that are not shared with Peruvian GBS from this study and those obtained by Ramos et al. (16) (light green). Also, some Peruvian GBS C. jejuni human genomes from this study and C. jejuni chicken genomes share a number of accessory genes, forming two different groups of strains inside the GBS cluster.

Chinese genomes have 105 exclusive genes; most of them related to prophages, emphasizing the presence of genes that encode type IV secretion system proteins like VirB2, VirB4, VirB9, VirB10, and VirB11, and *cpp* genes important for plasmid mobilization, as well as *cag* pathogenicity island proteins (Table S1). Peruvian Amazon strains have 49 accessory genes, but most of them encode hypothetical protein, according to the NCBI. However, they possess DUF domain proteins, endonucleases, and integrases that indicate the presence of a prophage inside genomes (Table S2). Finally, six Peruvian GBS C. jejuni genomes from this study and the four chicken genomes share 48 accessory genes, emphasizing the detection of type IV secretion system proteins like VirB7, VirB9, and VirB11 and other prophage proteins (Table S3).

**FIG 6:**
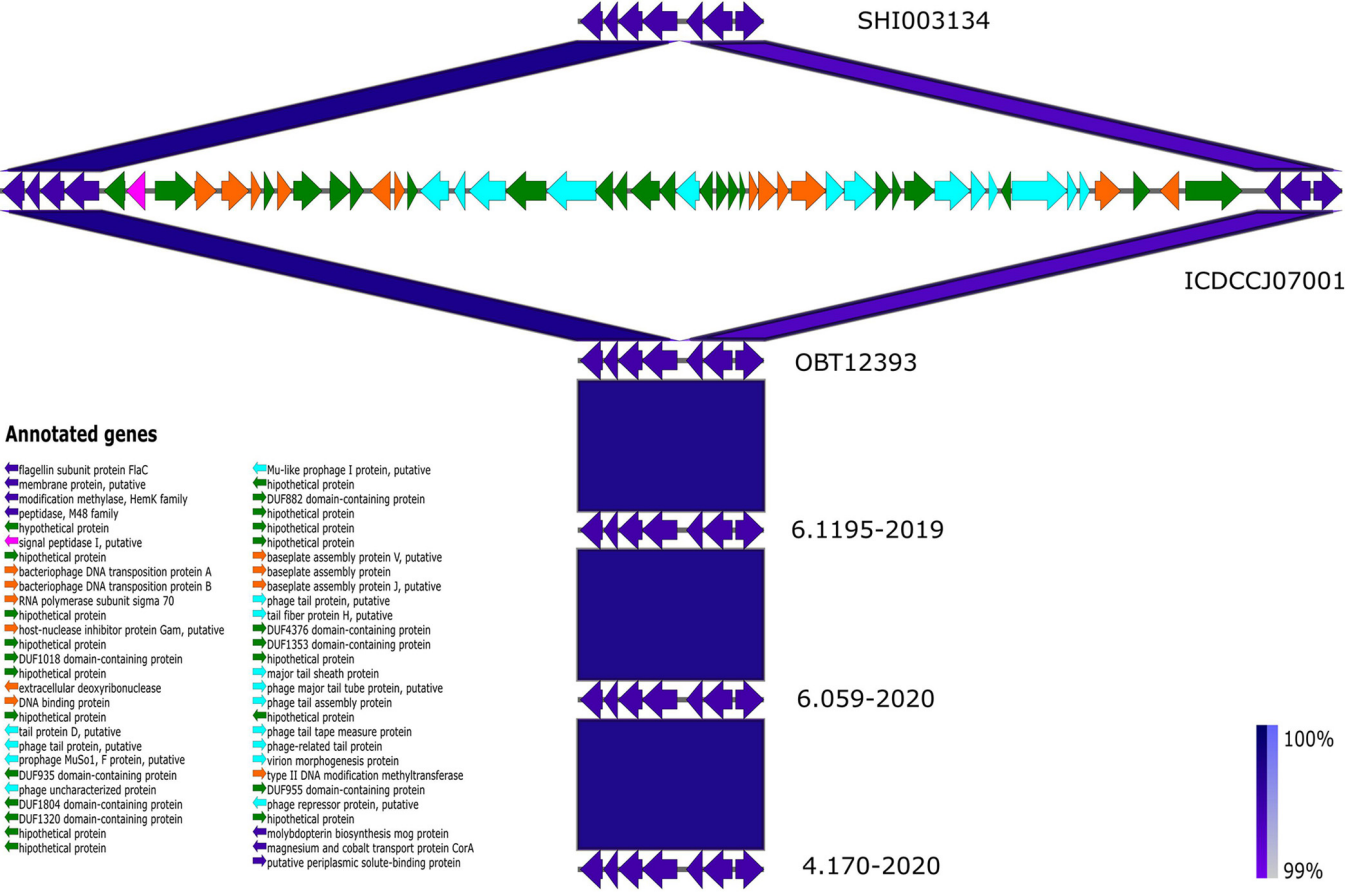
Genomic comparisons of Campylobacter Mu-like phage 1 region with Easyfig and the BLAST algorithm. The position of this region is compared between representative genomes. Arrows represent the locations of coding sequences, and blue lines reflect the degree of homology between pairs of phages. Colors mark specific predicted protein functions: bacterial proteins are in blue, DNA packaging/modification of nucleic acids is in orange, morphogenesis/lysis is in sky blue, signal peptidase is in pink, and hypothetical proteins are in green.

To have a better vision of the differences between the Chinese and Peruvian strains, the genomic regions corresponding to Campylobacter Mu-like phage 1 were extracted, using some strains representing the clades obtained in the phylogeny and comparative genomics, detecting that the prophage regions were absent inside the Peruvian genomes from all sources and studies evaluated (Fig. 6).

## DISCUSSION

Molecular epidemiology of pathogens is a very strong tool to allow investigators to identify at the molecular level the distribution and preventions of pathogens that cause disease that are difficult to track, like GBS associated with C. jejuni. The principal problem of connecting GBS with C. jejuni is that most patients develop symptoms like paralysis several weeks after a gastrointestinal infection and usually C. jejuni is not detected in stools (3). The rapid identification of these types of pathogens and the detection of possible sources of infections in an outbreak are important for planning strategies of prevention. Achieving knowledge of these two characteristics is not always easy; the process would be extensive and sometimes impossible to resolve, especially for syndromes that have more than one possible pathogen involved.

GBS has been reported several times in Peru since 2015, after few reported cases for many years (19). However, in 2018, a first increase of cases was observed in several regions of the country (20); the probable cause was found to be an enterovirus (21). Later, in 2019, a second abnormal increase of reports throughout the country, which began in the North Coast of Peru, prompted the declaration of a state of emergency. Nevertheless, this time C. jejuni ST-2993 was reported as the most probable pathogen, because it could be cultured and sequenced in several stool samples (15).

Peruvian health facilities have low training for isolating and detecting fastidious infectious agents in stool samples, like C. jejuni, a problem reported several years before (22), so it was very difficult to obtain a large number of isolates to evaluate and sequence. Despite this fact, an important number of C. jejuni isolates could be recovered in enriched media from human stool and chicken swabs. Considering the reduced viability of Campylobacter spp. in stool samples (18), using cultures to obtain C. jejuni strains in this study allowed us to recover 21 strains to sequence and include in the phylogenetic and comparative analysis.

All sequenced genomes presented a G+C% and a genomic size equal to other genomes of C. jejuni reported in previous studies (23–25). Identification using MLST detected the same genotype, ST-2993. This genotype, characterized initially as HS:41 according to the Penner serotyping scheme (26), had been reported before in some studies around the world in countries like Bangladesh (27), China (18), Belgium (28), and Peru (17), but few of them are connected to GBS cases and sequenced their strains to compare and analyze. Also, it is important to clarify that not only ST-2993 and CC-345 are unique genotypes related to GBS. There are other genotypes that were recovered from GBS patients’ stool, indicated as causing of GBS (27). However, the frequency of these genotypes inside the same geographic location and time is low compared to ST-2993, which was detected in several people in a short period of time and causing GBS (18).

The phylogenomics showed three divergent groups of genomes (Fig. 4). The first cluster is composed of Chinese strains from the 2007 GBS outbreak. It is important to emphasize that genomes obtained from this study included three strains with different patients’ characteristic: ICDCCJ07001 from a human GBS patient, ICDCCJ07002 from a human with gastroenteritis and diarrhea, and ICDCCJ07004 from an asymptomatic human (18). That is why there is a phylogenetic difference between them, even if they belong to the same year and location. The second cluster is made by Peruvian Amazon strains not related to GBS isolated from 2003 to 2005 (17). We could suppose that C. jejuni ST-2993 was silently circulating in the Peruvian Amazon for many years before the GBS outbreak, not being detected before because there is a great lack of diagnostic tools in those regions, like Loreto. However, genomic evidence of LOS locus genes like *cgtA*, *cstII*, *neuB*, and *neuC* inside C. jejuni in Peruvian Amazon children’s genomes suggests these strains always had the potential to cause GBS (10). The most internal clade included both human GBS and chicken C. jejuni strains from 2019 to 2020. An interesting fact is the detection of the same genotype inside chicken isolates. According to these findings, the current study reaffirms previous studies that chicken may be associated as a source of C. jejuni (9, 10, 18). Due to the very small number of ST-2993 chicken strains found and tested in this study, as well as the lack of sampling in other animal sources, it is necessary to perform more studies to confirm the principal animal source of C. jejuni linked to GBS. Zhang et al. (18) isolated C. jejuni strains from poultry samples taken from the GBS patients' houses, but the molecular typing of those strains obtained from human samples was different. In contrast, this study shows an epidemiological and genetic connection between clinical and chicken C. jejuni strains involved in GBS.

Other genes could help to understand the differences observed in phylogenetics. For example, resistance genes do not have the same distribution across isolates. Chinese strains have *tetO*, *cmeABC*, and *gyrA* mutation genes, while human and chicken C. jejuni strains in this study lack *tetO* but possess the other two determinants. On the other hand, Peruvian Amazon isolates only have *cmeABC*. The detection of *tetO* genes inside Chinese genomes is due to a resistance plasmid that confers tetracycline resistance (26). The wide distribution of *gyrA* mutation inside Peruvian isolates was reported in some studies before, especially in Lima (13). There is not enough data to confirm that quinolone resistance in Peruvian Amazon strains is less than in Lima, but those evaluated here do not have *gyrA* mutation. Notice that the three clusters have a notable difference related to resistance genes, but they are not the unique components that explained the difference between them. It is necessary to check inside every genome to find and understand other genomic differences between them.

Comparative genomics (Fig. 5) showed high clonality between C. jejuni ST-2993 isolates from different geographic locations and sources, detecting few exclusive genes on each genomic group (106 in Chinese, 49 in Peruvian Amazon, and 48 in Peruvian GBS and chicken genomes). This result is according to the study made by Kivistö et al. (29), which compares C. jejuni isolates from different sources, but the same genotype. About the differences between groups, Chinese genomes have a group of genes not observed in Peruvian genomes. Those accessory genes indicate the presence of plasmids and prophages inside these genomes. About this aspect, Zhang et al. (26) reported a tetracycline resistance plasmid of 44,084 bp inside one of these analyzed genomes, which contained *cpp*, *tetO*, and *virB* genes, important for plasmid mobilization, antimicrobial resistance, and virulence, respectively. All three genomes sequenced by these authors probably have that plasmid, because our data revealed the three Chinese genomes shared those genes (Table S1). Another interesting factor is the detection of cag pathogenicity island genes inside Chinese genomes, which could have been acquired by horizontal gene transfer (HGT) due to this unusual feature in Campylobacter strains, but it is very common in Helicobacter pylori, being an important virulence factor (30). This evidence could indicate the reason for the high virulence of Chinese isolates to cause an outbreak.

Peruvian Amazon genomes have another group of genes restricted to this group, but most of them do not indicate the presence of plasmid like in Chinese strains. However, more than 50% of these additional genes encode hypothetical proteins and do not have correspondence with genes or proteins inside the NCBI database. The rest of the genes are mostly recombination proteins (Table S2). In contrast, chicken isolates and some Peruvian GBS from this study share accessory genes that were identified using BLAST, detecting genetic content related to genomic elements like plasmids and prophages, some of them similar but not identical to those detected inside Chinese genomes Table S3. Parker et al. (31) indicate that accessory genes inside Campylobacter GBS genomes are related to capsule biosynthesis and LOS regions, but we detected other kind of genes exclusive from this group. However, it is necessary to carefully review hypothetical genes to determine their role and their transcendence in the evolution of this pathogen.

More than 50% of the 105 exclusive genes from Chinese genomes corresponded to parts of Campylobacter Mu-like phage 1 (CMLP1), which were not detected in any Peruvian genome. Previous reports mention CMLP1 could be part of inactive prophage remnants or be well adapted to Campylobacter species, but there is no evidence to clarify this statement (32). Also, a defined role for this prophage has not been established in the context of virulence (18). However, the Peruvian strains were pathogenic despite the absence of this prophage. Besides, LOS genes and other virulence factors are important components in the pathogenicity of GBS C. jejuni strains.

The main limitation of this work is related to the low frequency of recovering C. jejuni ST-2993 associated with GBS from stool samples. This happened because most of the samples received by the laboratory were collected several days before being processed, many of which were not transported to the laboratory in an adequate transport media like Cary-Blair using a cold chain. Also, C. jejuni is a fastidious microorganism, being affected by several factors like high oxygen concentrations, which reduce their viability, so the recovery rate of this bacteria decreases considerably after 24 h of transporting. Despite those situations, it was possible to recover a sufficient number of strains to determine the clonal relationship between C. jejuni strains from humans and chickens, as well as the performance of comparative phylogenetic and genomic studies. Besides, a new Peruvian Health Technical Standard (NTS N^o^ 175-MINSA/2021/CDC) was approved to regulate the epidemiological surveillance and laboratory diagnosis of GBS in order to avoid all the limitations mentioned before in a hypothetical future outbreak.

In conclusion, C. jejuni genomes ST-2993 obtained from GBS patients from 2019 to 2020 in Peru are similar in genetic content, and have a close phylogenetic relation, with chicken strains revealed by phylogeny and comparative genomics, pointing out these birds as a potential source of infection. Here, this study reports the emergency of a C. jejuni clone associated with GBS, which has spread and distributed in diverse regions of Peru. Also, the genotype ST-2993 has been circulating in Peru since 2003, based on Peruvian Amazon genome evidence, gaining genes that encoded the type IV secretion system to increase their virulence and cause an outbreak. Moreover, Peruvian GBS C. jejuni strains have the LOS locus responsible for causing GBS-like Chinese strains. These findings help to understand the emergence, dissemination, and evolution of this pathogen in Peru. It is necessary to encourage and reinforce the monitoring of public health pathogens that cause GBS using high-performance molecular biology methods like WGS.

## MATERIALS AND METHODS

### GBS epidemiology in Peru from 2018 to 2020.

The record of GBS cases reported by epidemiological week from 2018 to 2020 in Peru, collected by the Centro Nacional de Epidemiología, Prevención y Control de Enfermedades (33) of Peru, was plotted using Prism software v9.3.1 (GraphPad, USA) to evaluate the distribution of cases during this period of time.

### Study design and population.

A total of 1,040 stool samples (804 from 2019 and 236 from 2020; 818 clinical and 222 chicken samples), recovered under GBS laboratory surveillance by the Laboratorio de Referencia Nacional de Enteropatógenos of the Instituto Nacional de Salud (INS) of Peru from 2019 to 2020, were evaluated using FilmArray Gastrointestinal Panel (bioMérieux, France), but only the 99 positive stool samples for C. jejuni (92 clinical and 7 chicken samples) were included in this study.

### Ethical statement.

The study was developed within the framework of the project “Genomic Characterization of the Molecular Mechanisms of Antimicrobial Resistance and Virulence Factors in Clinical Isolates of Campylobacter spp. in Peru, 2010–2019” approved by the local Ethics Committee of the Instituto Nacional de Salud of Peru (D.R. 0154-2018-OGITT-OPE/INS).

### Isolation and identification of C. jejuni.

About 0.2 g of stool material was plated directly in Campylobacter selective agar (Oxoid, UK) using sterile swab sticks, incubating them at 42°C from 24 to 48 h under microaerobic conditions. The genus Campylobacter was confirmed by Gram staining observing the typical S-form, while the species was confirmed based on a biotype scheme for Campylobacter (34) composed of three tests including hippurate hydrolysis, hydrogen sulfide rapid production, and DNA hydrolysis. Molecular confirmation was performed by the PCR method developed by van de Giessen (35). Antimicrobial susceptibility to ciprofloxacin, tetracycline, and erythromycin was determined by disk susceptibility testing (36).

### Library preparation and whole-genome sequencing.

DNA extraction and purification was performed using 24-h cultures and DNeasy blood and tissue kit (Qiagen, Germany) following the manufacturer's instructions. DNA concentration (threshold: >100 ng/μL) and purity (threshold: 1.8 ratio of absorbance at 260/280) were evaluated by a spectrophotometer (Denovix, USA).

Subsequently, these were quantified in a Qubit 3.0 fluorometer (Invitrogen, Malaysia). The sequencing libraries were prepared using the Nextera XT library preparation kit (Illumina, USA), and genomic sequencing was performed using MiSeq reagent kit v2 (500 cycles) and a next-generation sequencer MiSeq (Illumina, USA) with a paired-end read chemistry (2 × 250 pb).

### Analysis of genomic data.

The quality of sequences was evaluated using FastQC v0.11.5. The low-quality adapters and nitrogenous bases were removed using Trimmomatic v0.38 (37). The sequences were assembled *de novo* using A5-miseq pipeline v20160825-2 (38). Genus identification and detection of possible contaminated contigs was carried out with Kraken v1.0 (39). Genotypes of strains were determined using the MLST program v2.11 (40) according to the next scheme: *aspA*, *glnA*, *gltA*, *glyA*, *pgm*, *tkt*, and *uncA*.

In addition, 4 C. jejuni genomes recovered in Lima from the same outbreak but for another study (16) were included in our analysis, as well as 5 genomes recovered in Loreto from symptomatic children not related to GSB (17). Moreover, 3 additional genomes from the GBS outbreak occurred in 2007 inside the province of Jilin, China (18): ICDCCJ07001, ICDCCJ07002, and ICDCCJ07004, identified as ST-2993, were also included in the phylogenetic analysis.

Also, in order to show all the Peruvian genetic diversity of C. jejuni, a maximum-likelihood phylogeny of Peruvian strains was inferred including all the previous genomes connected with GBS from this study (Ramos et al. [16], Pascoe et al. [17], and Zhang et al. [18]), but adding 84 Peruvian genomes belonging to other genotypes not connected with GBS from the study made by Quino et al. (41).

### Phylogenetic analysis.

The core genome alignment was performed with HarvestTools (42), taking as reference the Chinese strain ICDCCJ07001, which is the oldest strain related to GBS reported in the NCBI, and the SNPs were exported for downstream analysis. The phylogeny was inferred by the maximum likelihood method using RaxML v8.0 (43), applying the GTR+G model and 1,000 bootstrap. Removal of recombinant regions was performed using ClonalFrameML v1.11-3-g4f13f23 (44). The Microreact online tool (45) was used to visualize the results.

### Virulence factors of C. jejuni related to GBS.

The BLAST tool (46) was used to search genes located within LOS locus, such as beta-1,4-N-acetylgalactosaminyltransferase (*cgtA*), alpha-2,3 sialyltransferase (*cstII*), and N-acetyl neuraminic acid synthetase (*neuB* and *neuC*). Prediction of coding sequences for each library was performed using Prodigal v2.6.3 (47). Homologous genes of sequences were identified from a reference genome, with a BLAST algorithm with >90% identity and coverage >60% alignment to the reference. On the other hand, resistance genes were detected using the CARD database (48).

### Pan genome analysis.

The anvi’o v1.2.2 program (49) was used for the pangenome analysis of C. jejuni ST-2993. The anvi-gen-contigs-database script was used to create the matrix database to work on. Subsequently, the anvi-setup-ncbi-cogs script was executed to perform the annotation of the genomes, and finally the anvi-pan-genome script to perform the pangenome reconstruction. The results were visualized using the anvi-display-pan script. Accessory genes from each observed group obtained from this analysis were evaluated using BLAST. To evaluate the most important differences between genomes, the alignment regions were extracted and compared using Easyfig (50).

### Data availability.

All sequences obtained during this study have been deposited in the GenBank database (Bioproject: PRJNA719404).
