# Supplementary material for: Emergence and Molecular Epidemiology of Campylobacter jejuni ST-2993 Associated with a Large Outbreak of Guillain-Barré Syndrome in Peru
Source: Microbiol Spectr. 2022 Aug 16;10(5):e01187-22. doi: 10.1128/spectrum.01187-22 (PMC9603473; doi:10.1128/spectrum.01187-22)
Supplement: Supplemental file 1 — Supplemental material. Download spectrum.01187-22-s000.pdf, PDF file, 2.3 MB [file spectrum.01187-22-s0001.pdf]

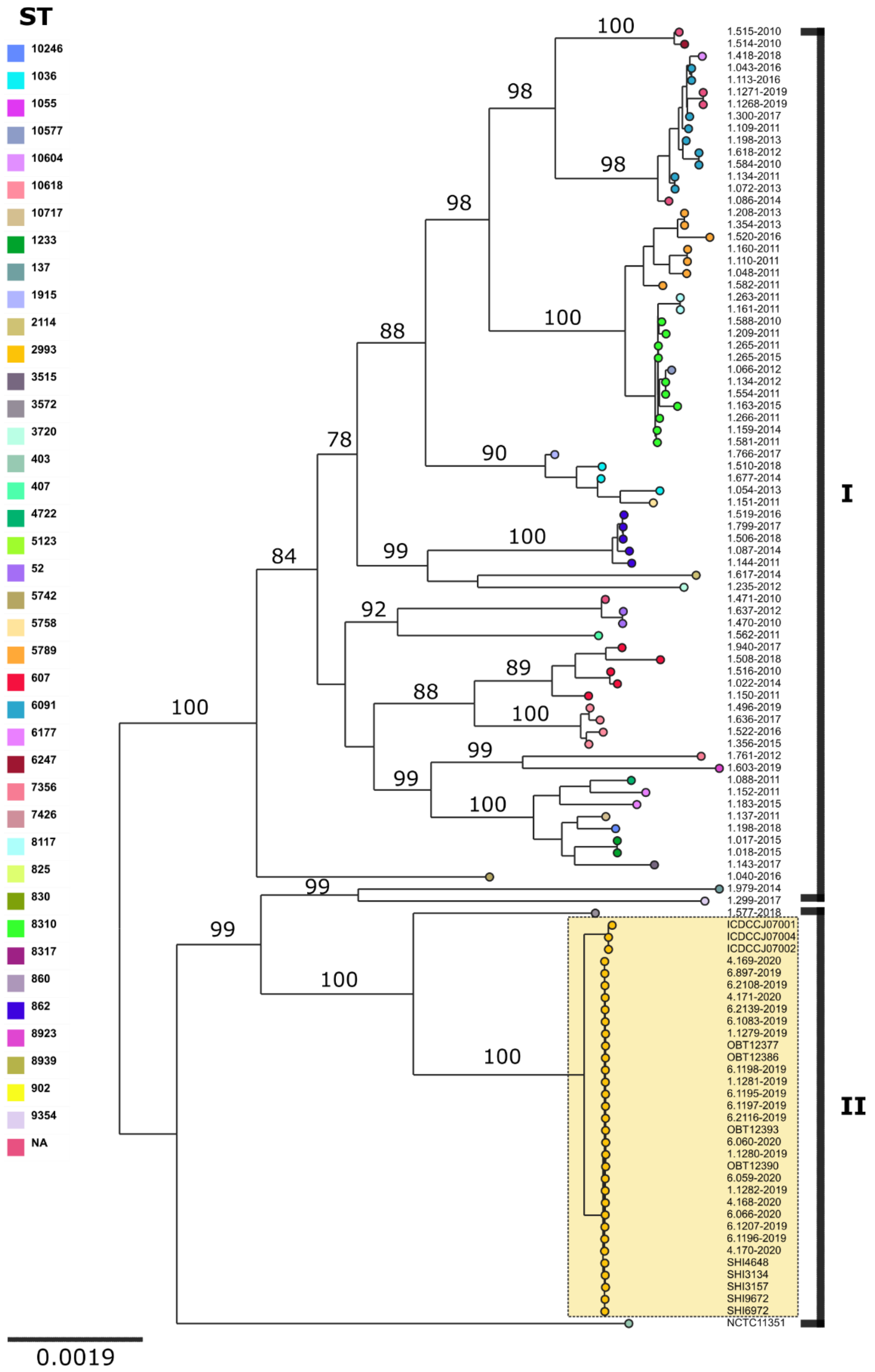

Fig. S1. Core genome maximum-likelihood phylogeny including *C. jejuni* strains which represent all genetic diversity detected in Peru. Branch lengths indicates substitution per sequence site. Bootstrap values calculated over 1000 repetitions are indicated at internal noted. Only bootstrap values above 80% are displayed. The name of each strain is indicated as a label parallel to the corresponding circles. The genotypes (ST) among the studied population are denoted by different colors. The clades are denoted by the numbers I and II. The strains used in this study are highlighted by a yellow box.

Table S1. Chinese GBS strains exclusive genes.

Number	Accession	Description	Max Score	Total Score	Query Cover	E value	Per. Ident	Acc. Len
1	WP_002807847.1	EexN family lipoprotein	159	159	100%	6.00E-49	100.00%	93
2	WP_032589444.1	LexA family transcriptional regulator	409	409	100%	6.00E-144	100.00%	224
3	EAJ2667066.1	ParA family protein	368	368	99%	3.00E-128	99.46%	186
4	WP_060806648.1	type IV secretion system protein	679	679	100%	0	99.70%	329
5	EAI4131472.1	hypothetical protein	171	171	100%	1.00E-53	97.73%	88
6	EAQ58641.1	phage virion morphogenesis protein, putative	146	146	100%	7.00E-44	100.00%	95
7	WP_010790813.1	phage tail protein	251	251	100%	4.00E-84	100.00%	124
8	WP_052782948.1	phage tail protein	651	651	100%	0	99.69%	325
9	MPP71362.1	hypothetical protein	416	416	100%	2.00E-146	100.00%	217
10	EHP2143705.1	hypothetical protein	217	217	100%	5.00E-71	100.00%	117
11	WP_052797184.1	hypothetical protein	179	179	100%	8.00E-57	98.88%	89
12	EAJ4780361.1	ImmA/IrrE family metallo-endopeptidase	374	374	100%	9.00E-131	99.45%	183
13	WP_002855011.1	DDE-type integrase/transposase/recombinase	934	934	99%	0	100.00%	691
14	TXE88505.1	cag pathogenicity island protein	291	291	100%	2.00E-99	99.31%	148
15	EAJ7750962.1	NTPase	395	395	100%	1.00E-138	99.51%	203
16	EAL1135958.1	hypothetical protein	112	112	100%	1.00E-30	98.25%	104
17	WP_010790803.1	tail sheath protein	815	815	100%	0	100.00%	397
18	ECH3381829.1	resolvase	402	402	100%	3.00E-141	99.51%	204
19	EAK1993514.1	single-stranded DNA-binding protein	289	289	100%	1.00E-98	98.57%	140
20	EAJ1833152.1	multidrug effflux MFS transporter	133	133	100%	6.00E-39	88.89%	81
21	WP_002875273.1	phage tail protein I	422	422	100%	3.00E-149	100.00%	210
22	EAK5144574.1	hypothetical protein	111	111	100%	2.00E-30	100.00%	77
23	WP_043012954.1	AAA family ATPase	1194	1194	100%	0	99.83%	597
24	ECQ6675420.1	hypothetical protein	291	291	100%	2.00E-99	99.31%	144
25	WP_002855090.1	major capsid protein	657	657	100%	0	100.00%	325
26	EGD3586268.1	TrbI/VirB10 family protein	799	799	100%	0	99.74%	390
27	ADT72445.1	hypothetical protein	78.2	78.2	100%	5.00E-18	100.00%	40
28	WP_087702801.1	tail protein X	126	126	100%	1.00E-36	98.41%	63
29	WP_013463365.1	pentapeptide repeat-containing protein	1421	1421	100%	0	100.00%	712
30	WP_002855241.1	hypothetical protein	685	685	100%	0	100.00%	335
31	WP_013463361.1	DNA topoisomerase 3	1393	1393	100%	0	100.00%	683
32	ECL6241221.1	hypothetical protein	226	226	100%	2.00E-74	99.12%	113
33	WP_013463358.1	relaxase/mobilization nuclease domain-containing protein	938	938	100%	0	100.00%	456
34	EAI9929485.1	hypothetical protein	523	523	100%	0	99.21%	254
35	WP_002854986.1	AAA family ATPase	624	624	100%	0	100.00%	307
36	WP_002854863.1	endonuclease	460	460	100%	1.00E-163	100.00%	223
37	WP_010790871.1	type IV secretion system protein	639	639	100%	0	100.00%	325
38	EAH6518036.1	hypothetical protein	119	119	100%	1.00E-33	100.00%	61
39	WP_002934973.1	Rha family transcriptional regulator	382	382	100%	5.00E-134	100.00%	188

40	WP_010790857.1	toprim domain-containing protein	829	829	100%	0	100.00%	408
41	WP_013463462.1	phage terminase large subunit	1139	1139	100%	0	100.00%	556
42	EAI6486306.1	RNA polymerase subunit sigma-70	124	124	100%	7.00E-36	100.00%	66
43	WP_002854846.1	hypothetical protein	642	642	100%	0	100.00%	320
44	WP_002795239.1	GPW/gp25 family protein	193	193	100%	6.00E-62	100.00%	96
45	WP_002854976.1	DNA adenine methylase	580	580	89%	0	100.00%	284
46	WP_002855254.1	phage tail assembly protein	129	129	100%	2.00E-37	100.00%	64
47	EFT8756555.1	TnpV protein	253	253	100%	8.00E-85	99.19%	124
48	EHN8349744.1	hypothetical protein	342	342	100%	4.00E-118	100.00%	184
49	WP_052802403.1	P-type DNA transfer ATPase VirB11	682	682	100%	0	99.70%	330
50	WP_010790861.1	DEAD/DEAH box helicase family protein	2140	2140	100%	0	100.00%	1935
51	SUW71662.1	cpp7	222	222	92%	1.00E-72	98.28%	123
52	EAI3389525.1	hypothetical protein	174	174	100%	2.00E-54	98.92%	93
53	WP_010790798.1	hypothetical protein	1263	1263	100%	0	100.00%	633
54	EAK8288189.1	hypothetical protein	175	175	100%	6.00E-55	98.90%	91
55	WP_002855209.1	hypothetical protein	461	461	100%	1.00E-163	100.00%	230
56	WP_032589440.1	hypothetical protein	246	246	100%	2.00E-82	100.00%	120
57	WP_010790809.1	DUF1320 family protein	305	305	100%	1.00E-104	100.00%	152
58	WP_087715680.1	hypothetical protein	176	176	100%	3.00E-55	98.86%	88
59	WP_010790804.1	baseplate J/gp47 family protein	768	768	100%	0	100.00%	388
60	EAB5236932.1	hypothetical protein	188	188	100%	3.00E-60	98.94%	94
61	WP_063674710.1	hypothetical protein	120	120	100%	4.00E-34	100.00%	63
62	WP_126235311.1	hypothetical protein	251	251	100%	5.00E-84	99.24%	132
63	EAH6204460.1	type I restriction endonuclease subunit R	47	47	61%	2.00E-04	95.45%	971
64	EAJ5453318.1	type II toxin-antitoxin system HicB family antitoxin	142	142	100%	1.00E-42	98.61%	72
65	WP_010790811.1	DUF935 family protein	912	912	100%	0	100.00%	456
66	WP_002935701.1	hypothetical protein	113	113	100%	2.00E-31	100.00%	54
67	WP_002922653.1	host-nuclease inhibitor Gam family protein	317	317	100%	8.00E-109	99.38%	161
68	EHQ6922440.1	hypothetical protein	196	196	100%	4.00E-63	100.00%	106
69	EAH6193234.1	hypothetical protein	159	159	100%	6.00E-49	100.00%	83
70	WP_010790808.1	hypothetical protein	342	342	100%	1.00E-118	100.00%	175
71	WP_002869703.1	hypothetical protein	375	375	100%	3.00E-131	99.47%	188
72	WP_002839671.1	TrbC/VirB2 family protein	171	171	100%	2.00E-53	100.00%	87
73	EIA45915.1	hypothetical protein	191	191	100%	4.00E-61	98.98%	98
74	ADR32204.1	conserved hypothetical protein	453	453	100%	2.00E-160	100.00%	233
75	WP_002855083.1	DUF4376 domain-containing protein	335	335	100%	8.00E-116	100.00%	168
76	WP_010790865.1	hypothetical protein	397	397	100%	4.00E-139	100.00%	204
77	WP_100621831.1	VirB4 family type IV secretion/conjugal transfer ATPase	1882	1882	100%	0	99.89%	922
78	WP_032600091.1	DUF1804 family protein	400	400	100%	2.00E-140	100.00%	208
79	WP_010790806.1	phage baseplate assembly protein V	424	424	100%	1.00E-149	100.00%	210
80	WP_002855275.1	phage tail protein	704	704	100%	0	100.00%	343

81	WP_002809052.1	hypothetical protein	214	214	100%	5.00E-70	100.00%	107
82	EAL9754729.1	type IV secretion system protein	440	440	100%	1.00E-155	100.00%	223
83	ADT66070.1	hypothetical protein	205	205	100%	1.00E-66	100.00%	106
84	EIB11362.1	hypothetical protein	59.7	59.7	100%	8.00E-11	93.94%	33
85	EAM0094322.1	S24 family peptidase	424	424	100%	3.00E-149	100.00%	228
86	EDO8457091.1	virion morphogenesis protein	120	120	100%	4.00E-34	100.00%	68
87	WP_010790858.1	hypothetical protein	608	608	100%	0	100.00%	301
88	WP_002876315.1	regulatory protein GemA	301	301	100%	2.00E-103	100.00%	149
89	EAL8887690.1	DNA-binding protein	188	188	100%	4.00E-60	98.94%	94
90	WP_002801138.1	vapd	278	278	100%	3.00E-94	100.00%	135
91	WP_002784658.1	phage major tail tube protein	344	344	100%	1.00E-119	99.41%	169
92	ADW85762.1	Mu-like prophage I protein, putative	541	541	100%	0	99.63%	271
93	WP_010790812.1	hypothetical protein	834	834	100%	0	100.00%	411
94	AAR29529.1	cpp46	520	520	100%	0	100.00%	266
95	WP_002790340.1	type II toxin-antitoxin system HicA family toxin	137	137	100%	1.00E-40	100.00%	67
96	WP_002804142.1	hypothetical protein	134	134	100%	1.00E-39	100.00%	66
97	ECO5369453.1	Tet(O)	1323	1323	100%	0	99.84%	639
98	ENI11260.1	hypothetical protein	272	272	100%	4.00E-92	100.00%	131
99	EAL7792366.1	hypothetical protein	105	105	100%	5.00E-28	98.11%	88
100	WP_002834097.1	P-type conjugative transfer protein VirB9	599	599	100%	0	100.00%	295
101	WP_002935694.1	hypothetical protein	418	418	100%	3.00E-147	100.00%	214
102	WP_002935694.1	hypothetical protein	418	418	100%	3.00E-147	100.00%	214
103	WP_010790801.1	phage tail tape measure protein	1461	1461	100%	0	100.00%	738
104	WP_010790750.1	AAA family ATPase	1005	1005	100%	0	100.00%	523
105	EAJ0379890.1	type IV secretory system conjugative DNA transfer family protein	1239	1239	100%	0	99.83%	603

Table S2. Peruvian Amazon strains exclusive genes.

Number	Accession	Description	Max Score	Total Score	Query Cover	E value	Per. Ident	Acc. Len
1	WP_002871222.1	hypothetical protein	433	433	100%	3.00E-153	100.00%	216
2	WP_002861640.1	hypothetical protein	549	549	100%	0	99.64%	279
3	WP_115766750.1	hypothetical protein	457	457	100%	6.00E-162	100.00%	231
4	WP_002861642.1	hypothetical protein	183	183	100%	3.00E-58	100.00%	92
5	EAC1315327.1	hypothetical protein	425	425	100%	4.00E-150	98.60%	215
6	WP_002861653.1	hypothetical protein	483	483	100%	6.00E-172	100.00%	243
7	WP_011049708.1	hypothetical protein	145	145	100%	1.00E-43	100.00%	74
8	WP_002896784.1	hypothetical protein	253	253	100%	1.00E-84	100.00%	126
9	WP_002875050.1	hypothetical protein	174	174	100%	1.00E-54	98.89%	90
10	WP_070210179.1	hypothetical protein	495	495	100%	1.00E-176	100.00%	245
11	WP_052796438.1	YqaJ viral recombinase family protein	572	572	100%	0	99.65%	284
12	WP_002865492.1	hypothetical protein	418	418	100%	4.00E-147	100.00%	215
13	WP_038402049.1	pentapeptide repeat-containing protein	358	358	100%	1.00E-124	100.00%	180
14	WP_002861657.1	hypothetical protein	201	201	100%	4.00E-65	100.00%	99
15	WP_002861666.1	hypothetical protein	250	250	100%	8.00E-84	100.00%	126
16	WP_087686048.1	hypothetical protein	313	313	100%	7.00E-108	99.35%	153
17	WP_002861675.1	hypothetical protein	273	273	100%	2.00E-92	100.00%	134
18	EBF5653809.1	hypothetical protein	276	276	97%	3.00E-93	99.28%	139
19	ECP8704265.1	hypothetical protein	1051	1051	100%	0	100.00%	512
20	EAI3872824.1	terminase	875	875	100%	0	99.77%	430
21	EDO8530879.1	hypothetical protein	285	285	91%	9.00E-97	97.95%	148
22	WP_002840259.1	hypothetical protein	185	185	100%	4.00E-59	100.00%	91
23	VEJ46701.1	hypothetical protein	446	446	100%	1.00E-157	95.28%	233
24	WP_002781524.1	hypothetical protein	174	174	100%	2.00E-54	100.00%	90
25	WP_002795630.1	hypothetical protein	263	263	100%	1.00E-88	100.00%	128
26	WP_052847758.1	site-specific DNA-methyltransferase	520	520	100%	0	100.00%	250
27	WP_002873428.1	DUF4376 domain-containing protein	407	407	100%	3.00E-143	100.00%	211
28	ECO2875125.1	NADH-quinone oxidoreductase subunit H	1164	1164	100%	0	99.65%	574
29	WP_000931940.1	hypothetical protein	129	129	100%	2.00E-37	100.00%	67
30	WP_052782368.1	DUF5309 family protein	702	702	100%	0	99.71%	344
31	ECP9225345.1	helix-turn-helix domain-containing protein	112	112	100%	8.00E-31	100.00%	66
32	WP_002840261.1	SocA family protein	341	341	100%	2.00E-118	100.00%	167
33	WP_057041419.1	hypothetical protein	646	646	100%	0	98.50%	333
34	WP_002875080.1	hypothetical protein	295	295	100%	1.00E-100	100.00%	149
35	WP_002875081.1	hypothetical protein	255	255	100%	2.00E-85	100.00%	127
36	EAH7544581.1	hypothetical protein	845	845	98%	0	99.77%	426
37	EIB82813.1	DNA/RNA non-specific endonuclease	454	454	100%	2.00E-161	97.77%	224
38	WP_052796437.1	hypothetical protein	150	150	100%	1.00E-45	100.00%	75

39	WP_052796436.1	hypothetical protein	159	159	100%	8.00E-49	100.00%	85
40	EAK5147796.1	phage regulatory protein	452	452	100%	1.00E-159	95.44%	243
41	WP_002861686.1	hypothetical protein	329	329	100%	1.00E-113	100.00%	163
42	WP_032582822.1	hypothetical protein	810	810	100%	0	99.26%	403
43	WP_002874357.1	hypothetical protein	165	165	100%	3.00E-51	100.00%	89
44	WP_002871234.1	hypothetical protein	134	134	90%	2.00E-39	100.00%	64
45	AZR08400.1	Prophage CP4-57 integrase	876	876	100%	0	99.31%	435
46	EAH7044186.1	hypothetical protein	1970	1970	100%	0	95.56%	1088
47	WP_002929400.1	hypothetical protein	494	494	100%	8.00E-176	100.00%	257
48	EAH7544586.1	hypothetical protein	1724	1724	100%	0	100.00%	880
49	WP_022552241.1	S24 family peptidase	487	487	100%	2.00E-173	100.00%	244

Table S3. Peruvian GBS strains exclusive genes.

Number	Accession	Description	Max Score	Total Score	Query Cover	E value	Per. Ident	Acc. Len
1	WP_011117583.1	hypothetical protein	256	256	100%	1.00E-85	100.00%	136
2	EAI3563774.1	hypothetical protein	203	203	100%	1.00E-65	99.05%	113
3	EAI3563778.1	PTS mannitol transporter subunit IIABC	249	249	100%	4.00E-83	99.24%	131
4	WP_216086201.1	hypothetical protein	235	235	100%	1.00E-77	100.00%	133
5	WP_053868691.1	hypothetical protein	254	254	100%	5.00E-85	100.00%	135
6	WP_011117588.1	type II toxin-antitoxin system PemK/MazF family toxin	261	261	100%	1.00E-87	100.00%	134
7	WP_203085004.1	CpaF/VirB11 family protein	647	647	100%	0	100.00%	316
8	WP_011799395.1	hypothetical protein	134	134	100%	3.00E-39	100.00%	73
9	EAI4085134.1	hypothetical protein	410	410	100%	3.00E-144	99.52%	209
10	EAJ0379438.1	hypothetical protein	208	208	100%	2.00E-67	99.07%	107
11	EAI7289806.1	ParA family protein	444	444	100%	2.00E-157	99.55%	222
12	WP_057039943.1	hypothetical protein	223	223	100%	1.00E-73	100.00%	111
13	WP_002815407.1	hypothetical protein	278	278	100%	6.00E-94	100.00%	143
14	WP_011117567.1	hypothetical protein	266	266	100%	2.00E-89	100.00%	137
15	EAJ5429143.1	type IV secretion system protein VirB9	726	726	100%	0	99.72%	356
16	WP_002815835.1	hypothetical protein	114	114	100%	7.00E-32	100.00%	56
17	EAJ2316235.1	type IA DNA topoisomerase	1311	1311	100%	0	99.85%	655
18	EAK2514780.1	hypothetical protein	158	158	100%	1.00E-48	98.75%	80
19	WP_002815556.1	hypothetical protein	174	174	100%	1.00E-54	100.00%	89
20	WP_203085002.1	type IV secretion system protein VirB7	82	82	100%	2.00E-19	100.00%	42
21	WP_011117559.1	hypothetical protein	238	238	100%	8.00E-79	100.00%	121
22	WP_011117554.1	hypothetical protein	108	108	100%	2.00E-29	100.00%	56
23	WP_014517461.1	hypothetical protein	644	644	100%	0	100.00%	318
24	WP_153605101.1	toprim domain-containing protein	821	821	100%	0	99.76%	410
25	WP_203085006.1	DUF1738 domain-containing protein	595	595	100%	0	100.00%	293
26	EFU4837091.1	hypothetical protein	222	222	100%	8.00E-73	100.00%	116
27	WP_053871798.1	AAA family ATPase	1669	1669	100%	0	100.00%	822
28	WP_203085007.1	type IV secretion system protein	771	771	100%	0	100.00%	382
29	WP_011799398.1	hypothetical protein	193	193	100%	1.00E-61	100.00%	101
30	WP_004306057.1	hypothetical protein	122	122	100%	4.00E-35	100.00%	61
31	WP_203085011.1	hypothetical protein	485	485	100%	1.00E-172	100.00%	246
32	WP_014517448.1	ribbon-helix-helix domain-containing protein	265	265	100%	3.00E-89	100.00%	135
33	EDO8520909.1	hypothetical protein	355	355	100%	2.00E-123	100.00%	186
34	WP_053871721.1	hypothetical protein	281	281	100%	3.00E-95	100.00%	142
35	WP_053871794.1	hypothetical protein	115	115	100%	6.00E-32	100.00%	66
36	WP_011117574.1	hypothetical protein	712	712	100%	0	100.00%	373
37	WP_011117560.1	single-stranded DNA-binding protein	315	315	100%	2.00E-108	100.00%	152

38	EAH4727589.1	hypothetical protein	540	540	100%	0	100.00%	261
39	EAJ1312062.1	virulence protein	455	455	100%	2.00E-161	99.56%	225
40	WP_053871795.1	DNA type IV secretion system protein ComB10	763	763	100%	0	100.00%	377
41	EAJ1312062.1	virulence protein	455	455	100%	2.00E-161	99.56%	225
42	EDP4184429.1	RepB family plasmid replication initiator protein	555	555	100%	0	99.64%	278
43	EAJ0273636.1	hypothetical protein	321	321	100%	2.00E-110	100.00%	166
44	EHP1578654.1	hypothetical protein	195	195	100%	1.00E-62	100.00%	103
45	WP_203085005.1	type IV secretory system conjugative DNA transfer family protein	1293	1293	100%	0	100.00%	629
46	WP_011799396.1	relaxase/mobilization nuclease domain-containing protein	1009	1009	100%	0	100.00%	523
47	WP_011117585.1	hypothetical protein	239	239	100%	1.00E-79	100.00%	121
48	EAH6004608.1	hypothetical protein	142	142	100%	2.00E-42	98.70%	77
